# Building a learning health care community in rural and remote areas: a systematic review

**DOI:** 10.1186/s12913-024-11194-7

**Published:** 2024-09-02

**Authors:** Shabnam Asghari, Jennifer Bent, Ali Modir, Alison MacDonald, Alison Farrell, Cheri Bethune, Wendy Graham

**Affiliations:** 1 Department of Family Medicine, Faculty of Medicine, Newfoundland and Labrador, Centre for Rural Health Studies, Memorial University of Newfoundland, 300 Prince Philip Dr, St. John’s, NL A1B 3V6 Canada; 2 Faculty of Medicine, Memorial University of Newfoundland, Newfoundland and Labrador, St. John’s, Canada

**Keywords:** Learning health care community, Learning health system, Rural health, Community engagement, Community participation

## Abstract

**Background:**

A Learning Health Care Community (LHCC) is a framework to enhance health care through mutual accountability between the health care system and the community. LHCC components include infrastructure for health-related data capture, care improvement targets, a supportive policy environment, and community engagement. The LHCC involves health care providers, researchers, decision-makers, and community members who work to identify health care needs and address them with evidence-based solutions. The objective of this study was to summarize the barriers and enablers to building an LHCC in rural areas.

**Methods:**

A systematic review was conducted by searching electronic databases. Eligibility criteria was determined by the research team. Published literature on LHCCs in rural areas was systematically collected and organized. Screening was completed independently by two authors. Detailed information about rural health care, activities, and barriers and enablers to building an LHCC in rural areas was extracted. Qualitative analysis was used to identify core themes.

**Results:**

Among 8169 identified articles, 25 were eligible. LHCCs aimed to increase collaboration and co-learning between community members and health care providers, integrate community feedback in health care services, and to share information. Main barriers included obtaining adequate funding and participant recruitment. Enablers included meaningful engagement of stakeholders and stakeholder collaboration.

**Conclusions:**

The LHCC is built on a foundation of meaningful use of health data and empowers health care practitioners and community members in informed decision-making. By reducing the gap between knowledge generation and its application to practice, the LHCC has the potential to transform health care delivery in rural areas.

**Supplementary Information:**

The online version contains supplementary material available at 10.1186/s12913-024-11194-7.

## Background

*Community participation *is known as the collective involvement of people in assessing their needs [1]. Prior research suggests that community participation in community services and programming is integral to the health of the community and its sustainability, and such participation can yield positive long-term health outcomes [2]. Community participation in primary and rural health care services has promoted more accessible and relevant services [2, 3], and it can result in higher community-member satisfaction with health services [4]. There is a long tradition of community-member contributions to various health services and preventative health programs [5–7].

A Learning Health care System (LHS) is a model that ‘draws from the best scientific evidence while tailoring optimal care to a local health care setting to each patient’ [8, 9]. As technology advances, so does access to clinical and person-specific data that can inform health care decision making [8]. An LHS has three core components: (1) foundational elements; (2) care improvement targets; and (3) a supportive policy environment [9]. Foundational elements include upgrading digital technology to collect data and facilitate data sharing within the health care system. Care improvement targets assist learning and health through clinical decision support tools, patient-centered care, and clinician-community links. A supportive policy environment includes financial incentives that reward high-value care, encourages transparency within the health care system and commitment from leaders. An LHS can lead to many optimal health care characteristics, including engaged patients, appropriate decision supports, aligned governance, and sharing of necessary data; however, adopting an LHS approach has come with an obstacle, to effectively engaging community members to achieve optimum health outcomes.

A Learning Health Care Community (LHCC) model expands on the LHS model by combining the core elements of an LHS with a fourth component: active and continuous stakeholder and community engagement to improve the quality and value of health care within a community [9]. An LHCC focuses on health care beyond a health care system and extends beyond an LHS, which focuses on collaboration in health care. The LHCC uses the best practices of the LHS model with evidence-based approaches to engage community members.

The aims of an LHCC include increasing collaboration between public health and traditional health care providers and intending to address all areas required to achieve optimal impact on health for the community. Collaboration from multiple community sectors and effort from the community is vital to the success of the LHCC. Previous literature has found that engaging community members in meaningful conversations regarding their health care is associated with improved health outcomes, quality of time, and better health care experiences [9].

The LHCC should be trusted and valued by all stakeholders and consist of an economically governable system while being responsive to community needs. Past involvement in discriminatory practices committed against vulnerable and minority populations has impacted community-institution relationships [10]. However, ongoing commitment of investigators and research teams, treating community members as partners, working collaboratively, increasing skills in relationship building, and listening to diverse voices can overcome mistrust by health care providers and researchers [10, 11]. Co-development with communities can assure trustworthy and targeted implementation to address community needs [9].

An LHS model lacks community involvement and fails to consider community-based health care problems [9]. As a result, these communities lack control and power over their health care. An LHCC places the voices of communities at the center of their health care, empowers community members, and increases opportunities for health-related learning [8, 9]. The LHCC framework is relatively new, and, there is limited research on the impact of LHCCs in rural areas. The majority of existing research on LHCCs is completed in urban areas, or it is outcome-focused with less emphasis on the enablers and barriers that enhanced or hindered the implementation process. We sought to address this gap in existing knowledge by summarizing the barriers and enablers across all LHCCs implemented in rural areas. The current review can be used as a guide to develop more targeted and seamless approaches to develop an LHCC and achieve community health outcomes.

## Methods

### Research aim

The current paper aimed to summarize evidence on the facilitators and barriers that are involved in the LHCC implementation process for rural health care providers, researchers, decision-makers, and community members who wish to implement an LHCC.

#### Question of interest

What are the barriers and enablers to building a learning health care community in rural areas?

### Research design and information sources

We conducted a mixed-methods systematic review. The JBI methodology for mixed-methods systematic reviews was used to inform the entire systematic review. Thematic coding was completed according to the *JBI* convergent-integrated approach [12].

### Eligibility criteria

A search of English peer-reviewed published articles that were rural-based, health-related, and involved the implementation of an LHCC was undertaken. Inclusion criteria included the following:

- Studies must have been conducted in rural areas. For the purpose of this study, areas were considered rural if they were outside cities [1]. The research team also checked the author’s definition of a rural area used in the included studies.
- Community participation took place and it was related to improving the health services, health knowledge, or well-being of community members.
- Studies must have included at least one of the following stakeholders (researcher, health care providers, health system leaders, etc.), as well as a community or patient population.
- Co-learning took place by community members and stakeholders. Co-learning refers to a bi-directional learning process, where knowledge exchange occurs between those involved [13]. Health care providers, leaders, researchers or investigators learned from the community (e.g., their feedback) and community members learned about a health-related topic or health service from health care providers, leaders, researchers or investigators.

Articles were excluded if they were the format of a letter to the editor, a systematic review, an audit, or an editorial. Studies focused on an urban area or simply on an LHS rather than an LHCC were also excluded. All included studies in the review and articles deemed eligible were assessed and included in the review.

### Search strategy

Rural and primary health care experts were consulted to identify keywords and studies on LHCCs, community engagement and health care practitioners. Then, in consultation with a librarian, the search terms were tested and refined. Next, a Public Services Librarian from Memorial University of Newfoundland searched databases including Medline (Ovid), Embase (Embase.com) and The Cumulative Index to Nursing and Allied Health Literature (CINAHL; Ebsco) to identify potentially relevant articles up until May 2, 2023. See Additional File 1 for the search history for Ovid Medline. Results were imported into Endnote X9 for deduplication and then into Covidence for screening.

### Study selection and data collection process

Relevant titles and abstracts by database searches resulted in the identification of 8169 articles. After duplicates were removed, 8096 articles remained. Articles were screened independently by two trained authors using Covidence, an online software that streamlines the production of systematic reviews. If an abstract was deemed relevant, a vote of ‘Yes’ was assigned to the article, and if an abstract was deemed irrelevant a vote of ‘No’ was assigned to the article. If both reviewers assigned a vote of ‘Yes’ the article moved into the full-text phase, and if both reviewers voted ‘No’, the article was excluded. Any abstracts that received conflicting votes or were assigned a vote of ‘Maybe’ were discussed and resolved between authors. After a consensus was reached, full texts of the remaining articles were obtained and this process was repeated. A calibration exercise was administered to portray the validity of the research on 10% of the articles in each stage and our approach was adjusted if it was required. For the abstract, 816 articles (10%) were randomly selected, and a third reviewer examined these abstracts. Reference lists and citations within full text articles were checked for eligibility. No additional articles were added using this approach. The same process was used for screening and data extraction. Next, the research team confirmed the included articles following review and conflict resolution. Weekly meetings were executed to discuss the articles. If there were any conflicts in article decisions, a third reviewer was involved in the resolution.

### Data synthesis methods

Included articles were moved to data extraction by both independent reviewers. A data extraction tool was developed using Microsoft Excel and pilot tested on 5 articles. Both reviewers independently entered the data to the tool. Columns were included that summarized key information pertaining to the article (i.e. Geographical Setting, Study Objectives, Community Group, Enablers and Barriers, etc.).

Data from each article was compiled into the Microsoft Excel sheet and reviewed by the research team. The compiled articles (*n* = 25) were reviewed by several team members and discussed during meetings until we reached consensus. Similar text fragments were grouped and categories were identified. Consistent with the *JBI*mixed-methods systematic review convergent-integrated approach [12], categories were identified based on assembled data from both qualitative and quantitative studies. Next, categories were aggregated to form themes relating to the barriers and enablers to building an LHCC. A consensus on the final list of themes was reached through group discussions to reduce bias and ensure consensus was reached.

Enablers and barriers were discussed in relation to the four main components of the LHCC. The Stakeholder and Community Engagement component highlights studies that identified effectively engaging community members to be pivotal to an LHCC success. Infrastructure for health related data-capture was relevant to studies that found harnessing contemporary technology, information sharing through in-person and online interventions, development of educational materials, and health-related data sharing to be vital to patient health. Care Improvement Targets were included as various stakeholders aimed to improve health, disease management, and increase awareness and uptake of prevention interventions within the community. Care Improvement Targets were organized according to the Institute of Medicine’s report: “Crossing the Quality Chasm: A New Health System for the 21st Century” six aims for success (1) safe; (2) effective; (3) timely; (4) patient-centered; (5) efficient; and (6) equitable [14]. A Supportive Policy Environment was included as acquiring proper funding, resources and support was deemed essential to the LHCC.

### Quality appraisal

The quality of each study was assessed independently by two authors using the Mixed Methods Appraisal Tool (MMAT) scoring system [15]. The average of each reviewer's assigned score determined the quality of each article. Studies of 75% and above were considered good quality, 50–74% were considered fair, and 50% and below were considered low quality. All studies that met inclusion criteria were included, despite the quality appraisal score.

## Results

**Fig. 1:**
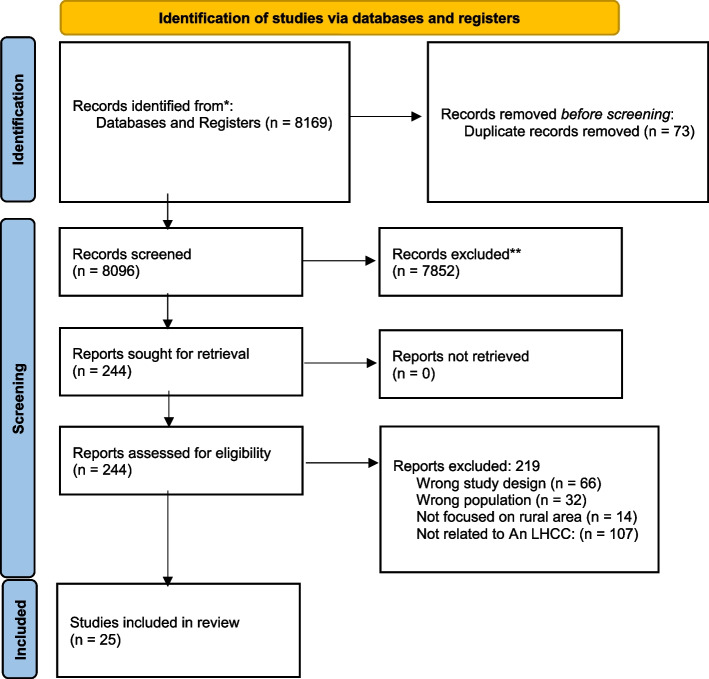
Prisma flow diagram

A literature search identified 8169 articles to screen and assess for eligibility. Following screening and the application of inclusion and exclusion criteria, 25 articles were included (Fig. 1).

Studies were conducted in the following countries: the United States (*n* = 15), India (*n* = 3), Canada (*n* = 2), Australia (*n* = 2), Guatemala (*n* = 1), Thailand (*n* = 1), and the Republic of Congo (*n* = 1). The characteristics of included studies are summarized in Table 1. All studies included stakeholder populations (as defined above) that experienced a form of learning health outcome in their community. There were no age-specific studies, and children were not exclusively excluded. Six articles (24% of all articles) were deemed fair, and 19 studies (76% of all articles) were deemed good quality. The studies were a mix of qualitative (*n* = 14), quantitative (*n* = 3), quality improvement (*n* = 2), and mixed methods (*n* = 6).

**Table 1:** Characteristics of included studies

	**First Author (year)**	**Country (Geographical Area)**	**Study objectives**	**Initiator**	**Study design**	**Data collection method**	**Stakeholders**	**Quality assessment**	**Sample size**
1	Arcia (2016) [16]	New York, USA	To collaborate with community members to develop tailored infographics that support comprehension of health information, engage the viewer, and may have the potential to motivate health-promoting behaviors	Columbia Community Partnership for Health	Participatory Research	Surveys, Voice record, Hand vote	Washington Heights and Inwood community members	75%	102
2	Kunz (2017) [17]	Santa Cruz County, Arizona USA	This case study describes the program components and key lessons learned from implementing Vivir Mejor! (Live Better!), a program tailored for the rural, Mexican American population	Organizers of the Diabetes prevention and management program	Case Study	Workshops, Digital Story Screening	The rural, Mexican American population	75%	136 patients for education sessions 137 participants attended nutrition sessions and 243 evaluated for HbA1c
3	Carpenter (2018) [18]	Unspecified, USA	To advance the practice of patient and family-centered care in hospitals, promoting medication therapy management for at-risk populations, and reducing non-agent emerg services	AHRQ Health Care Innovations Exchange	Program Evaluation	Mixed-Methods	Learning community members, project staff	75%	3 Learning Communities
4	Chhabra (2018) [19]	Punjab, India	To test the feasibility of a peer-driven intervention model in cervical cancer prevention in India	Two Northern India Universities	Program Implementation and Evaluation	Surveys, Workshops	Regular community members who identified as women aged 18–50	91.7%	68
5	Fung-Kee-Fung (2018) [20]	Ontario, Canada	To describe a system approach to redesign care, enabling timely access of patients suspected of lung cancer to a centralized specialty service while creating the dynamic adaptability to address both clinical and operational challenges	The Ottawa Hospital and the Ottawa Health Transformation Model	Program Implementation and Evaluation	Semi-structured Interviews, Stakeholder Mapping	Community of practice was established to engage stakeholders	75%	68 Key Influencers
6	Key (2018) [21]	Michigan, USA	To draw on lessons learned from decades of community-engaged health research and practice locally in the Midwest Region, and at the national level addressing issues related to genomics	Flint Community Based Organization Partners & National Community Committee of the CDC’s Prevention Research Centers	Community-based Participatory Research	Continuum Model	Flint Community Based Organization Partners, Patient-Centered Outcomes Research Institute, National Community Committee of CDC's prevention research centers	66.6%	–
7	Myers (2018) [22]	Philadelphia, USA	To describe components of a health system learning community and describe a learning community strategy that involved forming multiple teams to address cancer screening and disparities in 2 health systems	Thomas Jefferson Health Team and the Lehigh Valley Health Network	Program Implementation	Quantitative data collection, Focused interviews	Diverse patients, health care providers, health systems leaders, public and private payers, and other stakeholders	83.3%	73 Interviews
8	Gierisch (2019) [23]	North Carolina, USA	To explore views, barriers, resources, and perceived values of engaging patient advisors in a national program of evidence synthesis research	Veteran Affairs Evidence Synthesis Program (ESP)	Qualitative	10 Interviews & 2 Focus Groups	ESP leaders (directors, associate directors) and programmatic staff, research assistants	91.7%	
9	Murray (2019) [24]	Boston, USA	To make the medical and patient communities aware of an Autism Learning Network that is based on the Institute of Medicine's definition of LHS	Autism Intervention and Research Network on Physical Health	Program Implementation and Evaluation	Quantitative data using a web portal, Document reviews and Observations	Anderson Center team, parents, clinicians, researchers, and data analysts/biostaticians	75%	12 Network Sites
10	Baba (2020) [25]	Republic of Congo, Africa	To identify strategies that can help to attract, support, and retain midwives in the fragile and rural Ituri province	Three Health Districts	Qualitative Participatory Research	Workshops & Phone Interviews	Female midwives, decision makers, managers	91.7%	49
11	Curtis (2021) [26]	Manitoba, Ontario, British Columbia, Saskatchewan, Canada	To further collaborative efforts to improve access to preventive health care for kidney patients and their caregivers using culturally safe practices	Local Indigenous community health care stakeholders	Participatory Action Research	Workshops, Modified Delphi	Male and female patients, caregivers, Indigenous peoples, researchers, and policy makers	66.6%	30
12	Donahue (2021) [27]	Arizona, USA	To describe the organization of the Epilepsy Learning Health care System (ELHS), a network that aims to improve care outcomes for people with epilepsy (PWE)	Epilepsy Centers and Community Services Organizations	Quality Improvement	Analytic Tools	Patients and family partners, providers, researchers, epidemiologists, and other leaders	66.6%	–
13	Golden (2021) [28]	Unspecified, USA	To examine a proof-of-concept project to develop processes within the WH-PBRN for rapid data collection to address queries from operations partners, while also returning impactful information to participating sites	Veterans Affairs Women’s Health Practice-Based Research Network	Program Implementation and Evaluation	Surveys, Administrative Data, Debriefing Notes	Women veterans, their providers, and their care settings	83.3%	1191 Women Veterans
14	Irby (2021) [10]	Southeastern USA	To understand how CEnR has been conducted and to identify needs to support CEnR within an emerging academic learning health system	Wake Forest School of Medicine/Wake Forest Baptist Health	Qualitative	Semi-structured Interviews	Faculty and research associates	83.3%	18
15	Keck (2021) [29]	Ohio, USA	To describe efforts in ImproveCareNow, a CLHS improving outcomes in pediatric inflammatory bowel disease (IBD), to increase the number of patients and families creating and accessing IKK, and the challenges faced in that process	ImproveCareNow CLHS Framework	Program Evaluation	Trusted Messengers, Community Organizing and Digital Outreach	Patients and their families	75%	91 Care Centres
16	Beks (2022) [30]	South West Victoria, Australia	To use the CONSIDER statement to critically reflect participatory research undertaken in partnership with an ACCHO in the rural context and identifies lessons of value for future research	Aboriginal Community Controlled Health Organisations (ACCHO): Dhauwurd Wurrung Elderly and Community Health Service, Deakin Rural Health (University Department of Rural Health)	Participatory Research	Clinical Audit, Scoping review, Yarning: Sharing stories	Aboriginal Community Controlled Health Organisations Dhauwurd Wurrung Elderly and Community Health Service, Deakin Rural Health (University Department of Rural Health)	75%	-
17	Lindeman (2022) [31]	NPY, Australia	This participatory action research project aimed to improve service delivery for Aboriginal women from the Ngaanyatjarra Pitjantjatjara Yankunytjatjara (NPY) region in remote central Australia	Domestic and Family Violence Service (DFVS) of NPY	Participatory Action Research	Four two-day workshops	Senior Anangu women, Anangu DFVS Staff members, non-aboriginal staff members, psychotherapist	58.3%	
18	Marsh (2022) [32]	Ohio, USA	This project aimed to deliver patient-centered and equitable diabetes care services that were previously unavailable to underserved older patients, and to improve outcomes	The Diabetes COACH Team	Quality Improvement	Biweekly Community Health Worker home visits and diabetes self-management education to measure the outcomes for 12 weeks, Mixed-methods	Diabetes COACH Team, Community health workers, Nurse Practitioner, Adults aged 65 years or older with uncontrolled Diabetes Mellitus type 1 or 2	83.3%	12
19	Mishra (2022) [33]	Odisha, India	To identify and explore the contextual factors embedded in the current health care delivery process through community participation at the village level	All India Institute of Medical Sciences	Participatory Action Research, Qualitative	Community-centered Group Activities ( Participatory Learning Action Tool)	Resident doctors, medical social workers, community members, other local health care workers	83.3%	20
20	Oser (2022) [34]	Colorado, USA	This study aimed to use a community based participatory approach called ‘Boot Camp Translation’ to adapt an existing Diabetes Self-Management Education Support program for delivery in rural primary care for English- and Spanish-speaking people with diabetes	The High Plains Research Network Community Advisory Council and the University of Colorado and University of Utah	Community-based Participatory Research	10 Virtual meetings	English-speaking and bilingual Spanish–English–speaking members from the High Plains Research network Community Advisory Council, researchers from the University of Colorado and University of Utah	58.3%	-
21	Pitchalard (2022) [35]	Chiang Rai,Thailand	This study aimed to investigate the effect of a peer-training program for village health volunteers (VHVs) to improve chronic disease management among older adults in rural Thailand	The Research Team	Program Implementation and Evaluation	Three-day training workshop once every three weeks, Mixed-methods	The research team, nurse practitioners, village health volunteers, community members	83.3%	78
22	Pullyblank (2022) [36]	New York, USA	To use the Reach, Effectiveness, Adoption, Implementation, and Maintenance framework to evaluate the diabetes and chronic disease self-management programs offered through the ‘Living Well’ program	Living Well Project Team	Quality Improvement, Program Implementation and Evaluation	Six week chronic disease and diabetes self-management workshops, RE-AIM tools, Mixed-methods	Living Well project team, community-based organizations, caregivers, anyone living in a rural area with a chronic condition,	75%	600
23	Quraishi (2022) [37]	Haryana, India	To develop TB-related story content using community knowledge and experiences; use the digital storytelling for TB awareness and education; and assess the effectiveness of the storytelling in reaching its audience, increasing TB awareness, and subsequently increasing TB notifications	ZMQ Development (ZMQ), an organization based in Delhi, India	Program Evalution	Surveys, TB notifications, Discussions with ZMQ project staff, and with women visiting TB health centers	ZMQ project staff, individuals visiting TB centers in Punhana block, local leaders, women’s self-help groups, local ASHA workers, and other local health workers	83.3%	731 survey & 19345 patients screened
24	Gregg (2023) [38]	Guatemala, Central America	To understand if the Care Group Approach as applied in the Curamericas/Guatemala Maternal and Child Health Project in isolated rural mountainous communities in Guatemala produced evidence of empowerment among the female participants	Curamericas/Guatemala Maternal and Child Health Project Staff	Qualitative	Semi-structured individual and group interviews, Care groups	Ququilum and Jajhuitz and Paiconop Grande and Aldea Poza community members, Curamericas Maternal and Child Health Project staff, Care Group Volunteers, Promoters, Self-help group participants	83.3%	96
25	Niranjan (2023) [39]	Alabama, USA	To measure Lung Cancer Screening knowledge before and after receiving education delivered by community health advisors (CHAs) among high-risk individuals living in medically underserved communities of Alabama and to determine impact of psychological, demographic, health status, and cognitive factors on rate of lung cancer screening participation	The Research team	Quantitative	Questionnaires	Local county coordinators and community health advisors, community members over the age of 55	66.6%	100

### Defining a learning health care community

Descriptions and definitions of LHCCs were extracted from all included articles (Table 2). Most articles did not directly use ‘learning health care community’ terminology; however, all included key LHCC components.

**Table 2:** Definitions of LHCC and key barriers and enablers as identified in included articles

	First author (year)	Definition of LHCC	Community participation process	Type of rural health service development	Enablers	Barriers
1	Arcia (2016) [16]	LHCC implementation involves patient participation in the development of robust data utility, use of new clinical communication tools, and knowledge building through patient reported outcomes. Opportunities for patients and families to be engaged at all levels is an essential component to an LHCC	Participatory design sessions were held to elicit participants’ perceptions of the meaning of each design, whether the infographics would motivate them to change their health behaviors, their design preferences, and feedback for improvement	Development of tailored infographics that support comprehension of health information	Prolonged engagement with stakeholders and check-ins with members, peer debriefings, regular consultations with groups, carefully designed infographics to help patients engage with their own health data	Low levels of health literacy, agreement bias in group settings, financial constraints, difficulty in recruitment
2	Kunz (2017) [17]	Community resources and policies, in combination with coordinated health system functions, enable productive interactions between informed, activated patients and prepared, proactive practice teams. Productive interactions lead to better outcomes by improving clinicians’ treatment decisions, and patients’ understanding of their health and adherence to recommended treatment regimens or behavioral modifications for better health	Community health worker roles were designed to reach and support distinct populations. Multisector partnerships were made to support engagement through continuing education workshops and digital story screening to encourage referrals to the program	Vivir Mejor! (Live Better!), a diabetes prevention and management program tailored for the rural, Mexican American population	Funding, strong leadership, open communication	Lack of understanding of how to operationalize multi-sector partnerships Evaluation of how partnerships impact diabetes management, prevention, and the broader community
3	Carpenter (2018) [18]	An LHCC involves a collaborative environment that supports the adoption of health care innovations and motivates organizational change	LHCCs focused on adopting innovations in 3 high priority areas: (1) advancing the practice of patient- and family-centered care in hospitals, (2) promoting medication therapy management for at-risk populations, and (3) reducing non-urgent emergency services	The practice of patient and family-centered care in hospitals ; (2) Medication therapy management for at-risk populations; (3) Reducing non-urgent emergency services	Adoption of a learning community model, establishing a patient advisory council, maintaining partnerships, making incremental changes, strong communication, and achieving effective stakeholder engagement	Obtaining enough staffing resources, maintaining stakeholder commitment, and recruiting and training individuals
4	Chhabra (2018) [19]	A community health worker can conduct outreach to at-risk individuals in the community, provide needed education, and make linkages to care. Community members can act as health promotion catalysts, provide community education, encourage health promotion, and service navigation. They should have the opportunity to focus on issues that are important to them and can serve as leaders, influence health decisions, and have the skills to know how the community can instill change	Women referred their friends, family, and peers to attend a cervical cancer prevention workshop. Four focus group sessions with them were completed that involved the completion of a questionnaire	The Cervical Health Action and Intervention (CHAI)	Peers were educated as health advocates on how they could better promote healthy change in their communities, small, hands-on environment	Enrollment failure due to a lack of power to demonstrate efficacy and feasibility, lack of funding, limited in-person reach of rural populations and health care facilities
5	Fung-Kee-Fung (2018) [20]	Individual competence, systems thinking, cohesive vision, team learning, and the integration of different perspectives are elements of an LHCC. Also, there is a common goal of learning, a collective of multiple stakeholders with individuals outside of the hierarchy institution, that are considered equals is essential to LHCC development	A sample of health care professionals, patients, and caregivers were selected to represent the greatest variety of organizational and individual perspectives. This group was engaged through semi-structured interviews and stakeholder mapping	Application of an LHS paradigm to lung cancer care	A system open to ongoing innovation, dynamism of an LHS, continuous improvement in response to new evidence and information	Resistance to change, conflicting goals and priorities
6	Key (2018) [21]	Engagement of community at the organizational and institutional levels is a key element of an LHCC. Community was defined by identifying stakeholders one intends to engage and those working within and utilizing services of the LHS. Stakeholders within the LHS are critical to the knowledge that the health system produces and may include researchers, clinicians, insurance providers, and other key staff embedded in the system	**-**	**-**	Community/patient advisory boards participatory approach, stakeholder access to data, incremental improvements	Effectively engaging the community, funding, maintaining stakeholder engagement
7	Myers (2018) [22]	Multi Stakeholder engagement, shared value and mission, identification of the problem, evidence-based solutions commitment, and a sound operational approach are key elements of an LHCC	A coordinating team, a steering committee, and patient and stakeholder advisory committees were formed to address cancer screening and disparities in health systems	Colorectal Cancer Screening	Strong relationships with partners, having a shared mission	Maintaining long-term commitment from stakeholders
8	Gierisch (2019) [23]	An LHCC requires continuous stakeholder involvement and involves patient input throughout all levels of project implementation, training to ensure meaningful patient engagement, collaboration between patient populations and scientists, and the adoption of findings into practice	Qualitative interviews with researchers with different roles across 4 hubs and the coordinating center, and focus groups with veteran patients and caregivers were conducted. Patient informants were through ESP affiliated VA research centers	The Veteran Affairs Evidence Synthesis Program (ESP)	Shared goals, adequate training, obtaining appropriate resources, meaningful engagement use of time constraints	Patient representativeness, patient engagement, scientific complexity of evidence synthesis
9	Murray (2019) [24]	A Learning Network applies rigorous QI science methodology to identify gaps in services to be targeted for improvement and identify successful practices to replicate across the network site. LNs endeavor to achieve population health outcomes at scale. An LN platform does this by aligning clinical care, informatics, and culture to focus on continuous improvements, innovation, and research. Active partnering with patients and families in all aspects is essential	Various stakeholders, a part of the Anderson Center team, parents, clinicians, researchers, and data analysts/biostaticians met for an in-person, two-day design session followed by weekly collaborative leadership calls	The Autism Learning Health Network	Access to large data collection, Evidence-based solutions knowledge sharing	Effectively engaging participants, maintaining stakeholder engagement, funding, effective collaboration between stakeholders
10	Baba (2020) [25]	A participatory approach through a workshop methodology involves engagement of different stakeholders, builds on available data, reflection on evidence and real-world experience, promotion of LHSs and development of pragmatic strategies for the retention and attraction of health workers in rural settings	A workshop methodology was used in this study. Participatory workshops were held for community members and stakeholders. Discussions took place on the distribution and experiences of midwives	Recruitment and retention of health workers	Participatory/collaborative approach, strong communication between stakeholders, integration of workshops	Financial constraints, and poor financial planning, inequitable policy implementation
11	Curtis (2021) [26]	Collaborative efforts and meaningful engagement with patients, communities, and local health care stakeholders is essential to LHCC implementation as it allows for voices to be heard and incorporated into the project in a way that promotes shared decision-making and sustainability. Culturally safe clinical practices were identified as an essential component of the LHCC. All parties must share in the development and implementation of the project in a process that upholds mutual respect, learning, and dialogue	An engagement event on Kidney Check took place with a meal and presentations were completed to stakeholders. Community members shared their experiences managing kidney disease, their role within the networks, and insight on how to best use engagement strategies and local resources	Kidney Check Point-of-Care Testing	A patient-oriented approach, a multidisciplinary team, meaningful engagement with communities, collaboration	Lack of literature on recruitment in rural African American communities, low levels of health literacy, some participants were resistant to change
12	Donahue (2021) [27]	Using continuous Quality Improvement (QI) strategies to improve care over time is essential to LHCC implementation. Real-time data collection at the point of care, continuous measurement of outcomes essential to patients, patient engagement, evidence-based care, and standardization and improvement of care processes are components of the LHCC	A design session was completed to bring together clinical and community partners to refine the vision and mission for the network from a variety of perspectives	Establishing an LHS framework to improve health outcomes for individuals with epilepsy	Institutional Support, stakeholder engagement, shared experiences, incremental improvements, culture of respect, stakeholder collaboration	Obtaining appropriate resources, engaging participants in a hands-on manner, funding
13	Golden (2021) [28]	Interaction, collaboration, and synergies among researchers, clinicians, and educators, generating a mutually reinforcing relationship between research, practice, and policy are key elements of an LHCC. Real-time access to knowledge, engaged, and empowered patients are also important	Stakeholders were involved in the project development, implementation, data analysis and dissemination phases. Women completed patient feedback forms presenting for appointments in primary care clinics	Women’s Health Practice-Based Research Network embedded within Veterans Health Administration	Good communication with partners, continuous feedback, engagement, strong stakeholder collaboration, and broad dissemination of findings	Competing demands stakeholders, timelines
14	Irby (2021) [10]	Community is defined as any group affiliated by geographic proximity, special interest, health condition, or similar categories of shared identity. In an LHCC, community members and representatives from community organizations collaborate and share research roles with academic investigators and research teams. Relationship building, trust, open communication, co-learning, shared power, resources, and decision-making, mutual ownership of the processes and products of research, and community engagement are essential components	Professors and research associates of varied academic backgrounds and departments were identified and completed semi-structured interviews	**-**	Institutional support (e.g., funding, protected time, and respect), research resources (e.g., research centers), shared mission, strong partnerships, having an institutional review board	Potential for burnout and strain, time commitment, competing community and academic goals/priorities, community mistrust, sustaining partnership, lack of funding
15	Keck (2021) [29]	Collaborative Learning Health Systems (CLHSs) are communities of patients, families, clinicians, and researchers who can all act as improvers by collaborating to improve health care and health outcomes. CLHSs facilitate collaboration by supporting effective communication between patient advocates and providers, shared goal development, mutual responsibility, and accountability for the production and sharing of resources and information	Patients and families were asked to opt in to the CIRCLE eNewsletter that links patients and caregivers to resources, and learning sessions. One-on-one meetings were conducted to establish a relationship and aid in co-producing training materials	ImproveCareNow, a collaborative Learning Health System for pediatric inflammatory bowel disease	Demonstrating value of stakeholder engagement, collaboration, organization into affinity groups, engagement using a systematic approach	Participant recruitment, resistance to change, creating stakeholder connections, knowledge/resource sharing with community
16	Beks (2022) [30]	Establishing partnerships that can inform steps of the research project, based on epistemological rationale where community members can lead and guide the research. Using participatory and culturally appropriate approaches to engage participants and for them to lead the discussion on health	Community-based System Dynamics Method and the Indigenous research method of Yarning (Story-telling)	-	Community partnerships and involvement, using culturally appropriate research methods, and community-based participatory approaches, sharing the use and value of the research with participants, strong relationships and communication	A need for flexibility in the research plan, meeting timelines, maintaining commitment and engagement with partners
17	Lindeman (2022) [31]	The LHCC can be adapted to different culturally diverse contexts, to enable genuine intercultural learning, and draws on Aboriginal and non-Aboriginal knowledge. This LHCC recognises the potential to work with communities to support reinvigoration of kinship-centred protective relationships and the potential to develop language-based resources to support this strengths-based work	The LHCC used a participatory Action research process to support Aboriginal and non-Aboriginal participants to take part in the research. The iterative action learning cycles involved a series of four two-day workshops over almost two years	Enhanced Domestic and Family Violence Services	Knowledge of community, language, strong communication skills, strong relationships, trust, sharing of cultural knowledge and practices, participatory action research, collaboration, meaningful engagement, recognizing the value of participant feedback	Lack of knowledge and discomfort by non-Aboriginal participants, flexibility of researchers and research process
18	Marsh (2022) [32]	The LHCC involves evidence-based interventions to improve health outcomes, aims to increase accessibility to quality care for underserved communities and address challenges related to the social determinants of health. The LHCC involves community interventions integrated into the primary care setting, patient-centered programs, and strong communication between patients and health care providers	A Community Health Worker (CHW) visited every other week to participants' homes for 12 weeks. Patients were visited in tandem or individually. During their home visits, the CHWs performed their routine assessment and provided diabetes education. The CHW also facilitated the patient's video conference with a health care provider	Diabetes Care of Older Adults through Community Health Worker (CHW) and Telemedicine Access Model (Diabetes COACH TeAM)	Telemedicine, and community health worker involvement, collaboration, patient-centered approach, strong communication and relationships	Lack of resources
19	Mishra (2022) [33]	The LHCC is a ‘people driven’ activity, involving participation and planning at each level of governance, involving multiple stakeholders. The LHCC involves participatory learning action (PLA) that can build local capacity to work toward a healthier community. Communities instead of outsiders analyze their situations to ensure that any learning is translated into action	A team of resident doctors and medical social workers visited all the selected 10 villages and held discussions with key community members as well as health care workers. A facilitator led PLA exercises (i.e. chart making, resource map development) in 10 villages	A conceptual framework for community participation in rural health care	Community participation, active dialogue from political and administrative stakeholders, participatory learning action approach, needs assessment, leadership	Lack of clarity of the role of community participation, poor supervision, lack of resources, health illiteracy, pessimism among participants
20	Oser (2022) [34]	The LHCC involves partnerships with community members, academic researchers, and health professionals. Having a multidisciplinary team, strong communication, engaging community members and patients, and community-specific knowledge is vital to the LHCC	10 virtual meetings over a six-month period with Community Advisory Council members including 15 diverse community stakeholders took place. Ad hoc members were added to round out expertise and perspectives	Enhanced Diabetes Self-Management Education and Support program called ‘Diabetes One Day (D1D)’	Community-based participatory research approach, one-day LHCC structure, virtual education, inclusive recruitment strategies, adaptability, strong communication, development of educational materials	Lack of resources
21	Pitchalard (2022) [35]	The LHCC involves creating links between the health care system and the community, co-learning, collaboration, strong communication skills, and knowledge sharing. The LHCC promotes interpersonal support, and seeks to improve health management and performance of health care workers	Village health workers were recruited by nurses at meetings. Diverse stakeholders were engaged and they participated in focus group discussions and completed research questionnaires. They completed the peer-training program for three days per week for three weeks	Peer-training Program to improve chronic disease management among eldrly	Participatory approach, supportive environment, communication, stakeholder feedback, group discussions, strong relationships, stakeholder engagement, training based on community needs, noting the value of participants	Identifying core program activities
22	Pullyblank (2022) [36]	Multi-sector collaboration between a rural health care system and community-based organizations are essential to the LHCC. It involves continuous capacity-building efforts with community partners, the use of health care system assets, and a cycle of quality improvement. The LHCC aims to improve health and health outcomes	Caregivers, as well as adults with a chronic condition were invited to participate in the program. Also clinicians made referrals to patients who were eligible. Workshops consisted of 8 to 16 participants and took 2.5 h each	Living Well Program	Strong community-clinic linkages, leveraging health care system assets, strong communication, community partners, and engaged stakeholders, adaptability, buy-in from partners, dissemination of outcomes, collaboration, multi-disciplinary team, multi-sector approach	Recruitment, lack of coordination between community-based organisations, low health care system engagement and lack of funding, and limited staff
23	Quraishi (2022) [37]	The LHCC engages communities using a participatory approach with the aim to increase health knowledge. The LHCC involves collaboration between community members, health care providers, and heath experts. Also, community knowledge and experiences are used to create educational messages that can address knowledge gaps	Individuals visiting TB centers in Punhana block, local leaders, women’s self-help groups, local community health workers, and other local health workers were invited to voluntarily partake in the Story Labs. The Story Labs sessions included 20–25 individuals and were facilitated by staff using a discussion guide	Story Labs: Digital TB awareness- raising storytelling	Using a systematic process, collaboration, participatory approach, digital storytelling, communication, engaged participants	The systematic process is human resource- intensive
24	Gregg (2023) [38]	The LHCC can allow for sharing of theoretical and practical knowledge through social experiences with community members. Using a Care Group Approach can increase participation from the community and empower people to make positive health behavior changes for themselves and for their children and families. The LHCC can increase engagement in community activities for improved health and beyond, thereby enhancing social capital in the community	A Care Group composed of 5–12 women Care Group Volunteers met together every 2 weeks with a Promoter to learn one or a small number of health messages that they each shared with their neighbors. Volunteers would meet with the mothers of assigned households, either as a group or individually during a visit to the woman's home to share and discuss the message(s)	Curamericas Maternal and Child Health Project	Group interview approach, group learning, collaboration, strong communication, strong relationships, participatory approach, knowledge sharing	Lack of resources, time, and literacy and knowledge of volunteers
25	Niranjan (2023) [39]	The LHCC aims to collaborate with community health advisors to increase community health service knowledge and uptake of preventative health services. Strong relationships with community health workers, community involvement, trust and partnerships are vital to the LHCC	Local county coordinators and community health advisors recruited participants. Participants completed questionnaires and then underwent a single CHA delivered educational session that took about 30 minutes. Then, they completed the posteducation survey	Community Health Advisor Educational Initiative to Increases Lung Cancer Screening and Knowledge	Trusting relationships, structured educational intervention, collaboration, strong relationships, using existing infrastructure, partnerships, understanding of culture	Lack of knowledge of health guidelines, medical mistrust,

All LHCC projects aimed to improve health outcomes, such as disease monitoring, management and prevention, and knowledge sharing with community members. All involved collaboration between stakeholders, co-learning, integration of community feedback, and discussed the community participation process. Additionally, most articles discussed developing rural health services such as online learning or research health networks, community-based intervention workshops, learning health systems, and evidence-synthesis programs.

### Enablers and barriers to building an LHCC

Common themes emerged across articles. Theme descriptions were created based on the content of included articles and were reviewed by the research team. A consensus on theme names was reached at team meetings. *Meaningfully Engaging Stakeholders* referred to the use of tools, activities, or community-specific or creative strategies to grasp the interest of diverse stakeholders to be involved in the LHCC implementation process or to be active members within the LHCC. *Stakeholder Collaboration* referred to bringing together diverse stakeholders to work together to implement the LHCC or to achieve a common goal as members of the LHCC. *Using a Participatory Approach* was used to describe recruitment and participation of people who are impacted by the LHCC. *Strong Stakeholder Relationships* referred to the creation of strong interpersonal and mutually beneficial partnerships between stakeholders. *Knowledge Sharing* referred to learning about a health topic from in-person or online interventions, through the use or creation of knowledge-translation tools, health-related activities or training sessions, or through the use of technology. A *Multidisciplinary Team* referred to teams that included at least two different stakeholders with different levels of expertise, skills or experience. For example, certain teams included community members, clinicians, nurses, specific patient groups, or community health workers.

The barrier *Obtaining Adequate Funding or Research Support* referred to challenges to obtain sufficient funding, technology, or personnel to support the development of the LHCC or to sustain the LHCC. *Participant Recruitment* was used to describe challenges to recruit community members, family members, patients, or other stakeholders that could assist with the development of the LHCC or who serve as active members within the LHCC. A *Lack of Knowledge* referred to challenges regarding a lack of knowledge about a health topic or illiteracy within the community. This barrier also described a lack of knowledge of how to effectively engage or recruit stakeholders. *Maintaining Stakeholder Commitment* was used to describe challenges relating to sustaining involvement of LHCC stakeholders, or underestimating the ongoing effort required to promote long-term commitment from stakeholders.

*Competing Demands of Stakeholders* was used to describe situations when stakeholders within the LHCC had conflicting priorities and therefore, hindered the progress of the LHCC. This barrier was also used to describe when stakeholders struggled to determine a shared mission of the LHCC. *Time Constraints* were identified by stakeholders that were either unable to or struggled to meet specific deadlines during the LHCC development process, or those who underestimated the time required to build relationships and effectively engage stakeholders.

See Table 3 for the most frequently noted enablers and barriers and the corresponding number of articles. See Table 2 for more detailed information on the enablers and barriers reported in each article.

**Table 3:** Most Frequent Enablers and Barriers and the Corresponding Number of Articles

Enablers	Number of articles	Barriers	Number of articles
Meaningfully Engaging Stakeholders	15	Obtaining Adequate Funding or Research Support	12
Stakeholder Collaboration	13	Participant Recruitment	5
Strong Communication	12	Lack of Knowledge	5
Using a Participatory Approach	8	Maintaining Stakeholder Commitment	4
Strong Stakeholder Relationships	8	Competing Demands of Stakeholders	4
Knowledge Sharing	7	Effectively Engaging Participants	4
Multidisciplinary Team	6	Time Constraints	3

The most frequently noted enabler that facilitated LHCC implementation included meaningfully engaging stakeholders (*n* = 15). This finding ‘Stakeholder and Community Engagement’ is one of the four main components of the LHCC framework. One LHCC described by Curtis et al. [26] integrated patient engagement at all stages of their program by ensuring participant voices were heard and incorporated into strategy development. They developed a multidisciplinary team of specialists, Indigenous care providers, patient partners, and policymakers who collectively contributed in the development and implementation process of the LHCC. The LHCC involved shared decision making at all stages and community-member knowledge was leveraged to enhance culturally-safe care.

Other enablers mentioned in four or less articles included having a shared mission, incorporating incremental improvements, demonstrating the value of stakeholder involvement, having strong leadership, prolonging engagement throughout the project, using culturally appropriate research methods, conducting a needs assessment, getting continuous feedback from the community, and providing stakeholders access to their health data.

**Fig. 2:**
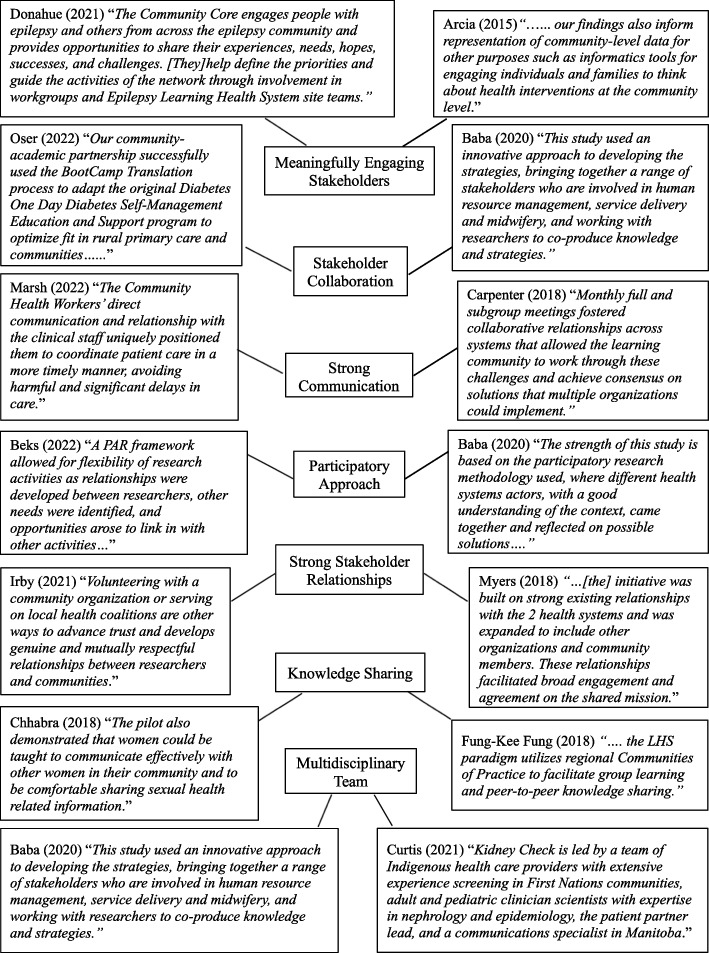
Mapping article quotes to LHCC enablers

Allowing patients to engage with their own health data to facilitate participation relates to the ‘Infrastructure for Health-Related Data Capture and Knowledge Sharing’ component of the LHCC model. For example, Arcia et al. [16] used a participatory-design approach where participants completed health surveys on their self-reported health outcomes and anthropometric measures. Data was returned to participants through clinical infographics and all participants effectively provided helpful feedback during design sessions. As a result, the LHCC led to tailored infographic designs that were more engaging, informative, and comprehensible according to participants. See Fig. 2 for quotes from included articles mapped to each enabler.

The most frequently mentioned barrier that hindered the success of LHCC implementation included obtaining adequate funding and resources (*n* = 12). This barrier relates to the ‘Supportive Policy Environment’ component of the LHCC framework. Having adequate funding (i.e. compensation for participants or stakeholders), or needed resources (i.e. technology, supplies, tools, or personnel) to meet LHCC deadlines was deemed essential for specific LHCCs.

**Fig. 3:**
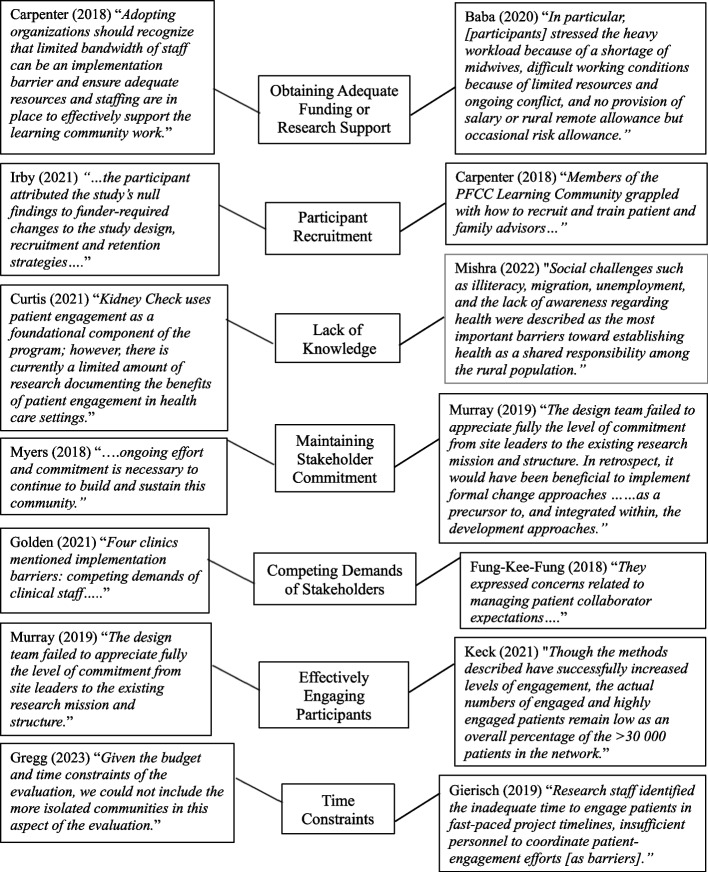
Mapping article quotes to LHCC barriers

Other barriers mentioned in one or two articles included mistrust from the community, achieving effective stakeholder collaboration, a need for flexibility in the research plan, resistance to change, stakeholder burnout, maintaining stakeholder connections, knowledge, and resource dissemination, having limited staff, and time commitment. See Fig. 3 for quotes from included articles mapped to each barrier.

Care Improvement Targets involve increased learning through clinician-community links, the use of clinical decision support, patient-centered care and optimizing delivery of health care and community-based resources [9]. Twenty studies (*n* = 20) reported on care improvement targets. Murray et al. [24] reported on the ‘Autism Speaks Autism Treatment Network’. This network leveraged community-partnerships to transition to an LHCC. The LHCC incorporated a model of co-production of knowledge between families and clinicians that promoted learning of each other’s expertise and values. They provided opportunities to take part in remote and in-person meetings, and participation in workgroups and monthly webinars. The LHCC resulted in growth of enrollment by almost 700 members. Among these studies (*n*= 20), enablers and barriers were described in alignment with the Institute of Medicine’s six aims of quality care [14].

- Patient-centered care: Seventeen studies (*n* = 17) involved the patient or family member involvement to improve patient health or patient-centered care (i.e. by consulting patients and implementing their feedback).
- Safety: Five studies (*n*= 5) reported safety of health care or research practices (i.e. the Kidney Check LHCC could increase the use of culturally safe practices in Indigenous communities) [26].
- Timely: Five studies (*n* = 5) reported that the LHCC has potential to led to more timely access to interventions, services, and resulted in faster decision-making by health care providers, and decreased wait times for patients.
- Efficient: It was reported in eleven studies (*n*= 11) that the LHCC could lead to greater efficiency (i.e. one study connected individuals who frequently use emergency services for non-urgent conditions to more appropriate care, resulting in fewer 911 calls) [18].
- Effective: Twenty studies (*n*= 20) reported on the effectiveness of the LHCC (i.e. one study developed a program that led to increased health knowledge and disease management strategies amongst affected populations) [34].
- Equitable: Seventeen studies (*n*= 17) reported on the equity of the LHCC. For example, one LHCC offered free health knowledge or exercise classes to vulnerable populations [33].

## Discussion

The current review aimed to summarize available evidence on the enablers and barriers of rural LHCC implementation. The review of LHCC studies suggests that LHCC implementation can increase community participation in planning health services and health-related activities. Health-related organizations implemented the LHCC to increase collaboration between community members and stakeholders, integrate community feedback in planning, and increase co-learning to expand knowledge sharing and improve health and services within the community.

Stakeholder engagement and collaboration were crucial enablers to LHCC success. It is unsurprising that stakeholder engagement was identified by multiple authors as active and continuous stakeholder and community engagement is a core component of an LHCC. Osborn & Squires [40] investigated perspectives on patient engagement using a survey in 11 countries: Australia, Canada, France, Germany, the Netherlands, New Zealand, Norway, Sweden, Switzerland, the United Kingdom, and the United States of America. They found that across countries patients who were more engaged with their health services, received greater quality care, and reported more positive views on the health care system. When stakeholders understand the value of the project, their involvement are provided with clear direction, and can interact with their data, they are more likely to be engaged. Also, the importance of stakeholder collaboration in health care is reiterated by Mullins et al [9] and supported by the Robert Wood Johnson Culture Health Framework [41]. According to this framework, achieving healthy equitable communities involves collaboration from multiple sectors to have the greatest impact on public and community health [9, 41]. Methods noted to facilitate collaboration include staying connected through various platforms (i.e. online networks, email or text-message), maintaining this contact on a regular basis, and meeting regularly as a group.

Although funding agencies have recognized the value of the LHS and LHCC models, obtaining adequate funding and resources was the greatest barrier identified in included studies [9]. Authors emphasized the challenge to receive robust investments from partner organizations and funding agencies to meet project staffing requirements, compensate participants for their time, and to purchase equipment and materials needed for the LHCC. In order to attract investors, it is important to document the benefits of the LHCC and present this information to organizational leaders and stakeholders. Furthermore, developing a feasible plan and demonstrating how you can sustain innovation can be influential for investors. Participant recruitment was also commonly documented as an obstacle. Particular solutions to address this issue have been noted in the included studies, such as participants being invited by a trusted and recognized expert in the field, putting effort into building relationships with participants, collaborating with them to determine clear goals, determine meeting frequency in advance, and have a clear decision-making process.

LHCCs aimed to either increase learning by community members about health topics (i.e. increase health literacy, interest in preventative health care) from experts within the field, researchers or health care providers, or they aimed to increase learning by stakeholders from the community or a particular community group (i.e. consulting midwives on how to retain them).

The current review outlines some overlap in enablers and barriers to urban or larger scale LHCCs. Similar to rural LHCCs, urban LHCCs also identify stakeholder collaboration, inclusion of multi-disciplinary teams, and knowledge sharing as enablers [42–44]. A lack of knowledge, and competing demands of stakeholders were also identified as barriers to implementation of urban LHCC implementation [42, 43]. Enablers specific to urban LHCCs include having access to population health data, access to technology that can analyze large-scale data, and conducting research to make comparisons and pinpoint trends in data [42, 44, 45].

Barriers identified in urban LHCCs included concerns around legal, ethical and policy challenges regarding patient health information and challenges relating to developing technological safeguards to protect the safety and security of patient health information. In addition, language barriers were also identified as a barriers to urban LHCCs [43]. Differences in enablers and barriers between urban–rural areas are related to the differences between rural–urban communities. For example, urban communities consist of much larger more diverse populations with greater access to patient health data and advanced technology.

Additionally, although having a multidisciplinary team is identified in both rural and urban LHCCs as an enabler, this term is defined differently between the communities. Rural LHCCs face challenges to recruit and retain their workforce. For example, only 8% of physicians in Canada work in rural areas [46, 47]. Therefore, rural ‘multidisciplinary teams’ are less likely to include specialists, epidemiologists, data-analysts, health-system leaders or policy-makers compared to urban LHCCs. Further, urban LHCCs place less importance on sustained community engagement. One reason for this may be due to challenges relating to the effort and time required to effectively engage larger populations. The small population size of rural communities may be more practical to engage for longer-periods of time.

Rural populations experience unique challenges compared to their urban counterparts. Rural-areas differ in terms of their geographic location, population size, weather, size of their workforce, and access to financial resources and health care services [48, 49]. Rural communities often have a greater proportion of elderly residents with chronic conditions [50], have limited access to health-information [51], and health care providers [52]. Despite these challenges, rural LHCCs provide an opportunity to address rural health needs. Driven by evidence, rural LHCCs place considerable effort into engaging the community in the LHCC implementation process. Shared-decision-making and co-production of knowledge are priorities of rural LHCCs. Due to workforce shortages, health-related education and training for community members appear to be pivotal in LHCC success. As evidenced by the current review, acquiring adequate funding to support LHCC implementation, meaningfully engaging stakeholders, and fostering collaboration are key components of successful rural LHCCs. Furthermore, LHCCs strive to increase health system transparency and accountability of participating entities to make improvements to health care [9]. By increasing awareness of community health problems, and clinical and financial support, the LHCC has potential to have a positive impact on community health.

The role of the LHCC is to improve the quality and efficiency of health care, therefore the LHCC must not overlook the impact of the social determinants of health (SDOH). Extensive research has emphasized the role of the SDOH on patient and community wellness, despite the quality of health care available [53]. For example, the United States spends an extensive amount of money on health care but is ranked last compared to other developed nations for a multitude of health outcomes, likely due to a lack of public health and social programs [54]. Despite the significance of the SDOH on patient health outcomes, the majority of included articles did not report on the SDOH. Future research should investigate the role an LHCC can play in addressing community and patient social risk factors. In addition, there was a lack of studies that focused on specific patient outcomes following LHCC implementation, and the impact of patient outcomes on the success of the LHCC. There is a need for greater research on LHCCs related to patient groups facing certain health diseases and evaluation of the impact of the LHCC on these health outcomes. Also, included studies did not report on the use of pragmatic clinical trials. Future research should investigate the role of clinical trials on LHCCs in rural areas.

Overall, there was repetition of several enablers and barriers mentioned in the included studies (i.e. effectively engaging stakeholders was mentioned in 15 studies and acquiring adequate funding was mentioned in 12 studies). However, there was also variability in barriers and enablers noted by authors mentioned in only one or two articles. For example, having strong leadership was mentioned as an enabler in two articles, and having limited staff was mentioned as a barrier in one article. One explanation for this could be that certain enablers and barriers were very specific to the LHCC being implemented. Therefore, enablers and barriers can vary based on the approach that is taken by implementers. Further, certain LHCCs may not have had one strong leader helping with implementation, and instead used a committee that struggled to make decisions. In this case, a strong leader would not be noted as an enabler. Nevertheless, this variation indicates that there is a lack of knowledge on the enablers and barriers that facilitate LHCC implementation in rural areas and this is an area for future research.

There were several strengths to the current study. First, this study expands on prior research by collecting the most common enablers and barriers that result from LHCC development and informs implementers of the most common obstacles that may be faced. Second, the approach taken to conduct the current review was closely aligned with recommendations [55]. Third, given the current circumstances of rural medicine and patient engagement, our research team deemed it imperative to conduct a deeper review of how to encourage stakeholders and patients to be involved in a collaborative partnership. This study is the fundamental step to a more thorough environmental scan.

The present study must be considered with limitations in mind. Although a minimum of two researchers and a librarian completed an exhaustive search for articles that met the inclusion criteria, there is a chance that some studies were missed or potentially miscategorized. Additionally, included studies differed across cultural, geographic, sociological, and geo-political boundaries across countries and this may impact the generalizability of our findings in different settings. Searches were limited to those in the English language and published articles. Articles published following the completion of the literature search were not included in this review. Therefore, this review may be subject to publication bias. Further, two studies noted that their findings may have been impacted by selection bias, as despite participation being encouraged in their respective LHCC, some participants did not choose to attend [23, 28]. Additionally, Golden et al. [28] recruited participants based on convenience, subjecting their findings to sampling bias and Gierisch et al. [23] opted to not record their interviews, acknowledging the potential for introducing social desirability bias and confirmatory bias. Although some articles assessed the effect of the LHCC following using pre- and post-implementation assessments, the chosen metrics varied across the studies and approximately half of included studies did not evaluate the impact of the LHCC. Therefore, the current study lacked a systemic approach and analysis of LHCC outcomes.

Ample evidence has shown that rural areas face poorer health outcomes than urban areas, due to a lack of infrastructure, health care providers, screening and medical equipments, and accessibility. The current study can be particularly useful to health care providers, researchers, health-related organizations, policymakers and leaders living in rural areas who want to reduce this health outcome gap, improve health-knowledge, patient engagement in health services planning, and research in their community. The current review summarizes the available evidence on the enablers and barriers to implementing an LHCC in rural communities and it is useful for anyone looking to implement an LHCC. Our findings highlight a clear lack of studies on rural LHCC implementation, as well as a lack of studies that evaluate the effect of the LHCC post-implementation, preventing the opportunity to conduct a meta-analysis. The LHCC model is relatively new, therefore, this review can increase awareness of the many benefits of this model and help to inform approaches to transition to this model.

## Conclusion

The LHCC is built collaboratively on a foundation of meaningful use of health data and empowers health care practitioners and patients in informed decision-making. Despite the number of barriers to implementing an LHCC, all studies that did implement LHCCs reported potential for positive outcomes. Although there are a variety of commentary, and perspective papers on LHCC development, this study adds to existing literature by summarizing the essential enablers and experienced barriers to facilitate LHCC implementation. Taken as a whole, an LHCC can be a potential solution to increase community engagement and collaboration between health care providers, researchers, decision makers and community members, and to mobilize resources in rural areas, and thereby lead to improved health services, health-knowledge, and health outcomes in these regions. Further research is needed on evidence-based approaches to effectively engage communities to be involved with their health care as well as the long-term outcomes of LHCC implementation in rural areas.

### Supplementary Information

Supplementary Material 1. Search strategy

## Data Availability

Not applicable.

## Abbreviations

LHS: Learning health system
LHCC: Learning health care community
