# Supplementary material for: Building a learning health care community in rural and remote areas: a systematic review
Source: BMC Health Serv Res. 2024 Sep 2;24:1013. doi: 10.1186/s12913-024-11194-7 (PMC11370021; doi:10.1186/s12913-024-11194-7)
Supplement: Supplementary file 1 — Supplementary Material 1. Search strategy [file 12913_2024_11194_MOESM1_ESM.docx]

**Additional File 1: Search Strategy**

Ovid MEDLINE(R) and Epub Ahead of Print, In-Process, In-Data-Review & Other Non-Indexed Citations and Daily <1946 to December 20, 2021>. This search was rerun on May 2, 2023.

1 (learn* adj2 (health or care or healthcare or wellcare) adj2 communit*).tw,kf. 67

2 (learn* adj2 communit* adj2 (health or care or healthcare or wellcare)).tw,kf. 84

3 "community health record*".tw,kf. 23

4 "community clinical linkage*".tw,kf. 33

5 1 or 2 or 3 or 4 174

6 exp Learning Health System/ 158

7 (learn* adj (health or care or healthcare or wellcare) adj (system* or capacit* or program*)).tw,kf. 843

8 6 or 7 885

9 communit*.ti,ab. 645163

10 Community Networks/ 7157

11 9 or 10 648630

12 (engag* or collaborat* or participat*).ti,ab. 925642

13 "shared decision making".tw,kf. 11066

14 Community Participation/ 18011

15 Community-Based Participatory Research/ 5167

16 Social Participation/ 3031

17 exp Stakeholder Participation/ 3214

18 13 or 14 or 15 or 16 or 17 38757

19 ("healthcare practitioner*" or physician* or doctor* or nurs* or "health professional").ti,ab. 980471

20 exp Physicians/ 160274

21 exp Health Personnel/ 568446

22 exp Primary Health Care/ or exp General practice/ or exp family practice/ 246651

23 (primary adj (health or care or healthcare)).ti,ab. 152538

24 19 or 20 or 21 or 22 or 23 1533588

25 (rural or remote).ti,ab. 229121

26 Rural Population/ 65469

27 exp Rural Health Services/ 13687

28 Rural Health/ 23771

29 Hospitals, Rural/ 5093

30 25 or 26 or 27 or 28 or 29 255107

31 8 and 11 138

32 11 and 12 98533

33 11 and 12 and 24 26090

34 18 or 32 128548

35 24 and 34 34554

36 33 or 35 34554

37 30 and 36 3558

38 5 or 31 or 37 3856

39 5 or 31 306
